# Droplet-on-demand tape drive and X-ray emission spectroscopy prototypes for time-resolved serial crystallography on VMXi at Diamond Light Source

**DOI:** 10.1107/S2052252526005658

**Published:** 2026-07-30

**Authors:** Pierre Aller, Juan Sanchez-Weatherby, Abbey Telfer, Robert Bosman, Nicholas Devenish, Philip Hinchliffe, Sam Horrell, Jophy Ip, Richard Littlewood, Andrew Male, Eva Gimenez Navarro, Urszula Neuman, Jos J. A. G. Kamps, David Omar, Laura Parkinson, Muthraj Pandi, Nico Rubies, James Sandy, Anastasya Shilova, James Spencer, Jonathan Spiers, John P. Sutter, Amy J. Thompson, Catherine L. Tooke, Ben Williams, Tiankun Zhou, Michael A. Hough, Allen M. Orville

**Affiliations:** a Diamond Light Source Ltd Harwell Science and Innovation Campus Didcot OX11 0DE United Kingdom; b Research Complex at Harwell Rutherford Appleton Laboratory Didcot OX11 0FA United Kingdom; c School of Biochemistry and Cellular and Molecular Medicine, Biomedical Sciences Building University of Bristol Bristol BS8 1TD United Kingdom; d Centre for Computational Chemistry, School of Chemistry, University of Bristol, BristolBS8 1TS, United Kingdom; e Department of Life Sciences and Centre of Evolution University of Bath Claverton Down Bath BA2 7AY United Kingdom; King’s College London, United Kingdom; University of Padova, Italy

**Keywords:** serial synchrotron crystallography, droplet-on-demand tape drive, X-ray emission spectroscopy, metalloenzymes, serial crystallography, protein structure, sample delivery, X-ray crystallography

## Abstract

Time-resolved X-ray crystallography is experiencing a resurgence, in part because of serial methods that readily allow scientists to create stop-motion movies of the macromolecular functions of photoactivation, enzyme-catalysed reactions and ligand-induced conformational changes triggering further downstream signalling events. While some reactions can be initiated with light, either naturally or using photocaged compounds, a more generally applicable approach is to mix microcrystals with reagents at varying time points prior to exposure to the X-ray beam. A powerful approach has been to combine droplet-on-demand ‘tape drive’ sample delivery with X-ray emission spectroscopy (XES) that correlates atomic structure with the electronic states of metal ions within the sample. To the best of our knowledge, such a combined methodology has not been deployed previously on a synchrotron beamline but has been restricted to X-ray free-electron lasers. Here we describe two independent prototype experiments along the development pathway to a combined droplet-on-demand diffraction and XES system on the microfocus synchrotron beamline VMXi at Diamond Light Source. We demonstrate the collection of a high-quality serial diffraction data set from microcrystals within droplets having a volume of hundreds of picolitres deposited on a moving tape. In separate experiments on VMXi, we collected XES data from microcrystals of a copper enzyme delivered using a high-viscosity extruder. Together, these results demonstrate the feasibility of combined droplet-on-demand serial crystallography and XES experiments using a third-generation synchrotron beamline.

## Abstract 2

We describe proof-of-concept experiments towards correlated serial synchrotron crystallography (SSX) and X-ray emission spectroscopy (XES) from microcrystals on a microfocus synchrotron beamline. A droplet-on-demand tape drive system delivers microcrystals to the beam within well separated droplets with a volume of hundreds of picolitres, while XES allows validation of the redox states of metals within protein crystals.

## 1. Introduction

The emergence of X-ray free-electron laser (XFEL) facilities motivated development of serial femtosecond crystallography (SFX) data collection strategies, including many sample delivery methods that are amenable to time-resolved studies (Barends *et al.*, 2022 ▸; Branden & Neutze, 2021 ▸; Chapman, 2019 ▸). Serial crystallography is becoming an increasingly popular tool for structural biologists, especially at synchrotrons where beamtime is typically more readily available than at XFEL sources (Aller & Orville, 2021 ▸; Orville, 2020 ▸; Pearson & Mehrabi, 2020 ▸). Third- and fourth-generation synchrotron beamlines, with high flux and microbeam capabilities, are particularly well suited to serial crystallography (Jaho *et al.*, 2024 ▸; Mehrabi, Schulz, Dsouza *et al.*, 2019 ▸). The advent of diffraction-limited lattices (so-called ‘fourth-generation’ synchrotrons) enables even smaller beam sizes and significantly greater flux densities. Several beamlines are now fully dedicated to performing serial crystallography, including ID29 at ESRF-EBS (Orlans *et al.*, 2025 ▸), T-REXX at PETRA III (Mehrabi, Schulz, Agthe *et al.*, 2019 ▸) and MicroMAX at MAX IV (Gonzalez *et al.*, 2025 ▸).

At Diamond Light Source (a third-generation source), two macromolecular crystallography beamlines are particularly suitable for serial crystallography, I24 and VMXi. The micro-focus beamline I24 has developed a world-leading flexible sample-mounting system that enables rapid switching between rotation and serial sample delivery, similar in concept to the MD3 diffractometer from Arinax (Grenoble, France). The key difference is that, in the MD3 system, changing between setups requires replacing a custom-made attachment, with all subsequent motions driven by the MD3 itself. In contrast, on I24 the changeover is quickly achieved (<10 min) by swapping components mounted on a kinematic base, and the motion required for fixed-target experiments is provided by independent positioning stages. This permits the beamline to operate different data collection modes, either a ‘pin’ mode or a ‘serial’ mode. Furthermore, I24 can use different serial sample delivery setups, either a fixed target or a high-viscosity extruder (Birch *et al.*, 2023 ▸; Jaho *et al.*, 2024 ▸). On the other hand, VMXi was designed for *in situ* room-temperature data collection from crystals within crystallization plates. Moreover, VMXi is also well suited for serial data collection since it incorporates a multi-layer monochromator (DMM) producing a broad energy bandpass (∼5 × 10^−3^ Δ*E*/*E*), resulting in high flux (>2 × 10^13^ photons s^−1^ at 16 keV) with a microfocus pink beam (10 × 10 µm) (Sanchez-Weatherby *et al.*, 2019 ▸; Sandy *et al.*, 2024 ▸). The characteristics of VMXi make it well suited to serial crystallography in terms of flux, beam size and detector repetition rate (Eiger2X 4M, running in continuous mode at 500 Hz).

Time-resolved crystallography studies typically require interpreting electron-density maps that represent mixed intermediate populations. This often presents different possible reaction intermediates at a certain time point, which may or may not be easy to resolve from the electron density alone. Consequently, complementary spectroscopic methods are incredibly valuable for time-resolved studies, since they can provide additional orthogonal data to help resolve ambiguities (Wilmot & Pearson, 2002 ▸). When studying metalloproteins it is possible to combine X-ray spectroscopy methods with X-ray crystallography. This can provide a critical advantage especially if/when the X-ray probed region simultaneously yields diffraction and spectroscopic information, because it removes uncertainty arising from data collection conditions (*i.e.* an offset in time or over differing exposures *etc.*). An important caveat is that X-ray spectroscopy ‘sees’ all the metal ions and may not differentiate between ordered and disordered species, whereas X-ray diffraction and the resulting electron-density maps reveal only ordered atoms (Einsle *et al.*, 2007 ▸; Sauter *et al.*, 2020 ▸; Zhang *et al.*, 2013 ▸). Combining serial crystallography with X-ray emission spectroscopy (XES) is therefore an elegant strategy for obtaining well correlated types of data from the same sample and X-ray pulse/exposure (Fuller *et al.*, 2017 ▸; Kamps *et al.*, 2024 ▸). Thus, XES data can help validate the interpretation of electron-density maps for catalytically active metal centres within metalloenzymes in time-resolved experiments.

An effective way to collect XES data while simultaneously collecting diffraction images uses a von Hamos geometry spectrometer. Developed for XFEL applications (Alonso-Mori, Kern, Gildea *et al.*, 2012 ▸; Alonso-Mori, Kern, Sokaras *et al.*, 2012 ▸; Kern *et al.*, 2013 ▸), a consortium including ourselves has demonstrated the drop-on-tape method (DoT) combining XES and diffraction experiments (Butryn *et al.*, 2021 ▸; Kamps *et al.*, 2024 ▸; Rabe *et al.*, 2021 ▸; Williams *et al.*, 2026 ▸). The DoT strategy, originally developed at a synchrotron facility as described previously (Roessler *et al.*, 2013 ▸), is now predominantly employed at XFELs (Fuller *et al.*, 2017 ▸). The main advantages of the DoT sample delivery system are (i) the low sample consumption, using nanolitre sample droplets ejected on demand and synchronized with the X-ray pulse, unlike jet techniques which also dispense sample between X-ray pulses, (ii) versatility of the solid support, allowing diverse reaction initiation methods such as turbulent mixing, gas diffusion and light illumination (Kamps *et al.*, 2024 ▸), and (iii) the possibility of exploiting anaerobic conditions (Lebrette *et al.*, 2023 ▸; Rabe *et al.*, 2021 ▸). The DoT system developed by the consortium at LCLS uses 2–5 nl droplets and has evolved into a data collection strategy that is complicated to install and operate, thus requiring significant support from the consortium. We have long noted that working with smaller droplets would significantly reduce sample consumption and improve the temporal resolution of time-resolved data.

Meanwhile, alternative tape-based sample delivery methods, such as those using capillaries to produce continuous sample streams, are being actively developed and used at synchrotron sources (Kamps *et al.*, 2024 ▸). The Center for Free-Electron Laser Science (CFEL, Hamburg, Germany) developed a simpler tape drive (CFEL TapeDrive 2.0), operating as a reel to reel system and using microfluidics to mix a microcrystal slurry with a ligand or substrate before depositing a streak of the mixture onto the moving Kapton tape (Beyerlein *et al.*, 2017 ▸; Henkel *et al.*, 2023 ▸; Prester *et al.*, 2024 ▸; Zielinski *et al.*, 2022 ▸). This tape drive is used on P11 (PETRA III) and MicroMAX (MAX IV); a similar tape drive exists on ID29 (ESRF-EBS). The main advantage of these systems is the ease of use, whereas a limitation is the lack of versatility for implementation of complementary methods.

*K*-shell XES is an element-selective technique which probes an atom’s electronic properties (*i.e.* redox and spin states) by measuring the energy dependence of emitted photons when an outer shell electron fills the *K*-shell hole created by the incident X-ray beam (Cutsail & DeBeer, 2022 ▸; Glatzel & Bergmann, 2005 ▸). For 3*d* transition metals the three most relevant emission lines are *K*α_1,2_ (2*p*→1*s*), *K*β_1,3_ (3*p*→1*s*) and valence to core (*K*β_2,5_) in decreasing order of their signal intensity. The *K*α_1,2_ doublet is a reporter of oxidation state, while *K*β_1,3_ is sensitive to metal–ligand covalency, spin state and oxidation state. The weakest (valence to core) signal can offer insights into the metal ion coordination environment, providing information on binding and the nature of valence molecular orbitals, but is also highly challenging to measure accurately due to its weak signal.

Combining time-resolved (tr) XES with serial crystallography has been successful in monitoring metal centres where the oxidation state correlates with enzyme reaction intermediates. In the iron- and oxygen-dependent isopenicillin *N*-synthase (IPNS), tr-XES with high-resolution SFX was measured from microcrystals co-crystallized with the tri­peptide substrate under anaerobic conditions and the reaction initiated with exposure to 100% O_2_ gas. This identified structural intermediates and conformational changes triggered by oxygen binding. The correlated XES monitored the width of the Fe *K*α_1,2_ peak and was consistent with the formation of the Fe^III^ valence state in the putative iron-bound superoxide intermediate and the associated structural changes throughout the enzyme that were revealed in the SFX data (Rabe *et al.*, 2021 ▸). In the case of methane monooxygenase, Fe *K*α_1,2_ XES data were used to verify the Fe^III^ versus Fe^II^ status of the dinuclear metal centre, which is very susceptible to photoreduction by X-ray exposure (Srinivas *et al.*, 2020 ▸). Time-resolved SFX and XES data from the P450 enzyme CYP121 from *Mycobacterium tuberculosis* catalysing a shunt reaction with peracetic acid revealed a ferric-hydro­peroxo intermediate in the 200 ms time-point data that is fully correlated with its 1.85 Å resolution atomic structure (Nguyen *et al.*, 2023 ▸). Finally, Mn *K*β_1,3_ tr-XES and tr-SFX were used to follow the S1 → S2 → S3 → [S4] → S0 transitions of the active oxygen-evolving complex in photosystem II triggered by pump–probe reaction initiation strategies (Ibrahim *et al.*, 2020 ▸).

Given these advantages, an important aspect for our tape drive development programme is its integration with XES. A challenge when working on metal-containing proteins is the possibility of site-specific X-ray radiation-induced perturbations, which can beneficially promote mechanistically relevant reactions, *e.g.* in redox proteins and enzymes, or lead to artificial redox state changes and experimental artefacts that may compromise the experimental outcome (Beitlich *et al.*, 2007 ▸; Chreifi *et al.*, 2016 ▸; Hajdu *et al.*, 2000 ▸). Serial crystallography helps to mitigate the X-ray induced artefacts in the sample by spreading the X-ray dose over typically thousands of microcrystals. Additionally, it can be challenging to identify conditions and time points where catalytic intermediates have built up to high occupancy. X-ray spectroscopy measurements from crystals provide complementary information, particularly when experiments are fully correlated such that different data types emerge from the same microcrystal volume. The additional data aid the interpretation of electron-density maps and refinement(s) of atomic model(s). Our goal is to exploit the XES signal on both synchrotron and XFEL beamlines using the same instrumentation at both types of facility.

Here we describe results from proof-of-concept experiments with prototype components for the DoT apparatus with the XES instrument under development on VMXi at Diamond Light Source. Serial crystallographic data measured from protein microcrystals within droplets of hundreds of picolitres (≤500 pl) on a moving Kapton tape are described. A second experiment reports XES results for copper *K*α_1,2_ measurements obtained from protein microcrystals using a four-analyser-crystal von Hamos spectrometer coupled with sample delivery using a viscous extruder. These results indicate the feasibility of carrying out this combined approach on a microfocus synchrotron beamline.

Our key design requirements were to keep the tape drive simple to use, transportable and compact, given the space constraints on VMXi, with the goal of portability to enable experiments on other beamlines. The prototype tape drive on VMXi demonstrated the ability to obtain good quality serial crystallography data at high resolution from microcrystals of the serine β-lactamase CTX-M-15. The von Hamos XES spectrometer prototype allowed representative measurements for Fe and Cu *K*α_1,2_ spectra of salts and of *Alcaligenes cycloclastes* copper nitrite reductase (*Ac*NiR) microcrystals. These results demonstrate that combined DoT/XES data collection is feasible at a third-generation synchrotron source, with further improvements expected from the diffraction-limited machine lattice at Diamond-II (https://www.diamond.ac.uk/Diamond-II.html) or at XFELs.

## 2. Methods

### 2.1. Sample preparation for tape drive experiments

Recombinant His-tagged CTX-M-15 (serine β-lactamase) was expressed and purified as previously described (Tooke *et al.*, 2019 ▸). CTX-M-15 microcrystal slurries were generated as previously described (Butryn *et al.*, 2021 ▸), using crystal seed stock that was generated by crushing macro-crystals grown by sitting-drop vapour diffusion (Tooke *et al.*, 2019 ▸); protein (5 µl at 20 mg ml^−1^) was mixed with crystallization solution [5 µl; 2.0 *M* (NH_4_)_2_SO_4_, 0.1 *M* Tris, pH 8] and seed (5 µl), and equilibrated against crystallization solution (500 µl) in 24-well sitting-drop plates (Hampton). Rod-shaped crystals grew within 24 h, with a maximum width of 3–8 µm and a length of around 8 µm. Crystal density was ∼1 × 10^8^ crystals ml^−1^ as measured using a TC20 automated cell counter (BioRad) or a counting chamber (Neubauer).

### 2.2. VMXi instrumentation setup and installation of tape drive prototype

**Figure 1:**
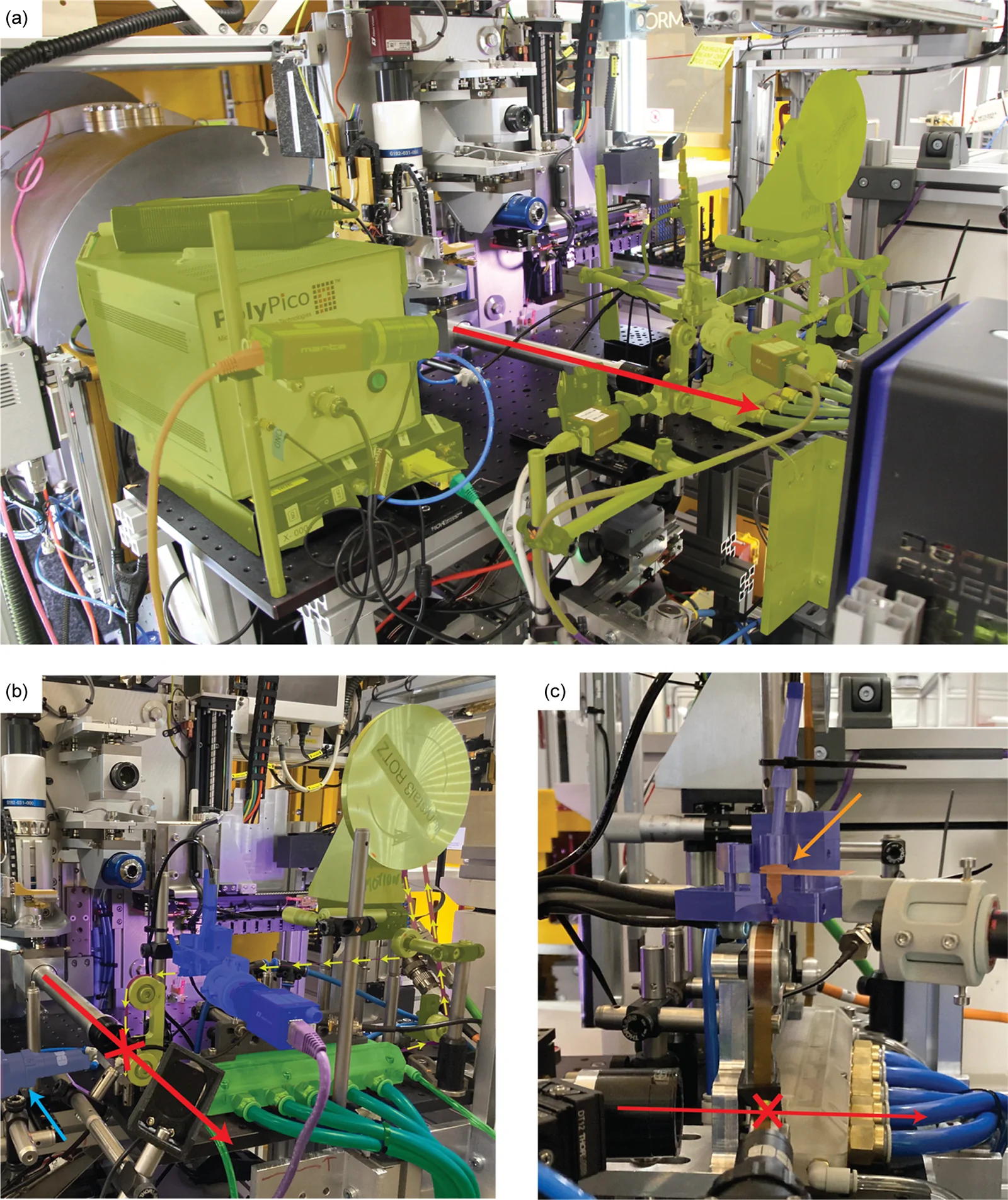
Tape drive prototype on VMXi. (*a*) Overall view of the installation of the tape drive prototype on the VMXi beamline. All the tape drive components are highlighted in yellow. The beam direction is given by the red arrow. (*b*) Focus on the tape drive prototype: mechanical components of the tape drive (motor and pulleys) are highlighted in yellow, the Kapton tape is highlighted in pink (with yellow arrows indicating the direction), the clean/dry unit highlighted in green, the piezoelectric injector (PEI) unit (camera, PolyPico mini-head and LED) highlighted in blue and orthogonal view camera highlighted in light blue (blue arrow). (*c*) Close-up view of the PolyPico mini-head highlighted in purple. The cartridge is highlighted in orange (orange arrow). The microcrystal slurry droplets are ejected onto the broad face and near the downstream edge of the Kapton tape, and are aligned to the X-ray interaction region (red cross) wherein the X-ray photon path is nearly parallel to the tape face.

The tape drive prototype consisted of a motor and tape, a wash–dry mechanism and a sample ejection system (Fig. 1 ▸ and Fig. S1). First, the system was installed and aligned to the VMXi X-ray beam path. The typical layout of the beamline has been described previously (Sanchez-Weatherby *et al.*, 2019 ▸; Sandy *et al.*, 2024 ▸). An aluminium breadboard (MB2060/M, Thorlabs) mounted on a MiniTec framework above the VMXi goniometer enabled the installation of the prototype tape drive as shown in Fig. 1 ▸(*a*). In standard VMXi operation, the X-ray beam is focused on the rotation axis of the beamline goniometer [Fig. S2(*a*)]. Due to the space requirements of the tape drive system, the sample interaction point for the experiments described here was instead placed 250 mm downstream from the standard position by modifying the settings of the microfocus bimorph mirrors [Fig. S2(*b*)]. An extension tube was designed to visualize the beam at the newly created reference point using a drilled lens (OPTEM 35-00-02-000, 4× magnification and 50 mm working distance) to allow the X-ray beam to pass through. In this way the X-ray beam could be focused onto a 50 µm YAG:Ce [yttrium aluminium garnet (YAG) activated by cerium; Crytur, Czech Republic] X-ray imaging screen at the new downstream sample position and the beamline on-axis viewing system could resolve the new focal point. The X-ray focal point and the interaction region of the tape drive system were aligned by referencing with the on-axis viewing camera. A second camera was placed orthogonal to the sample interaction region to allow continuous monitoring of the vertical and horizontal droplet position while collecting diffraction data.

#### 2.2.1. Droplet ejection system

Previous DoT tape drive systems used acoustic droplet ejection (ADE) (Hadimioglu *et al.*, 2016 ▸; Roessler *et al.*, 2013 ▸; Soares *et al.*, 2011 ▸) to dispense 2–5 nl droplets containing microcrystals onto a moving Kapton tape. To achieve lower sample consumption, we employed the piezoelectric injector (PEI) developed by PolyPico Technologies Ltd (Cork, Ireland) (Miniature Dispensing Head) (Butryn *et al.*, 2021 ▸). This device can dispense individual microcrystal-containing droplets in the range of 50 to a few hundreds of picolitres at a rate of up to 50 kHz. The reduction in droplet volume significantly decreases sample usage and will improve the signal-to-noise ratio, but it makes dehydration effects more prominent. To minimize dehydration, we positioned the ejection as close as possible to the interaction point (∼60 mm), with future designs incorporating a controlled humidity environment to mitigate such issues.

The droplet ejection module comprised (i) the PEI head and sample cartridge (used to hold the crystal slurry); (ii) the sample camera and the strobe LED (used to visualize ejection) and (iii) the wide-angle camera (used to monitor sample consumption) [Fig. 1 ▸(*c*)]. For longer duration experiments, the PEI cartridge was continuously topped up with fresh sample via a fused silica capillary (with inner diameter of 150 µm) using a remote-controlled precision 250 µl syringe pump (KDS Legato 130) sitting on a rocker to prevent sample settling in the syringe. Only the minimum amount of sample (10–20 µl) is maintained in the cartridge, thus preventing crystal settling and clogging of the cartridge aperture. The dead volume in the cartridge is approximately 7 µl.

#### 2.2.2. Tape drive and wash–dry mechanism

The prototype belt drive utilized a brushless DC motor (MAXON) to power the conveyor belt made of Kapton, 6 mm wide and 50 µm thick (kb RollerTech). The Kapton tape was treated with *Rain Repellent* (RainX) to make the surface hydro­phobic, thus improving drop adhesion and shape. The moving tape was cleaned after the interaction region by passing through a 3D-printed module designed to pressure wash and air dry the tape before it was re-injected with sample [Fig. 1 ▸(*b*)]. This device operates sequentially, performing two distinct stages of washing and drying. It uses compressed dry air and two independent distilled-water feeds, followed by an extended final drying step to minimize the risk of contamination. A comprehensive description of its internal mechanisms and performance will be provided in a forthcoming publication.

Although the tape drive can be operated at higher speeds, for this study we restricted the speed to 25 mm s^−1^ to ensure effective tape cleaning and sufficient residence time for microcrystals in the X-ray beam. This tape speed allows for a 720 µs maximum X-ray exposure, assuming an 8 µm crystal traversing the 10 µm X-ray beam, suitable for this application. In the final design we anticipate using a wide range of tape speeds (up to 1 m s^−1^) and therefore different exposure times on VMXi, as shown in Table 1 ▸.

#### 2.2.3. Synchronization and X-ray data collection

**Figure 2:**
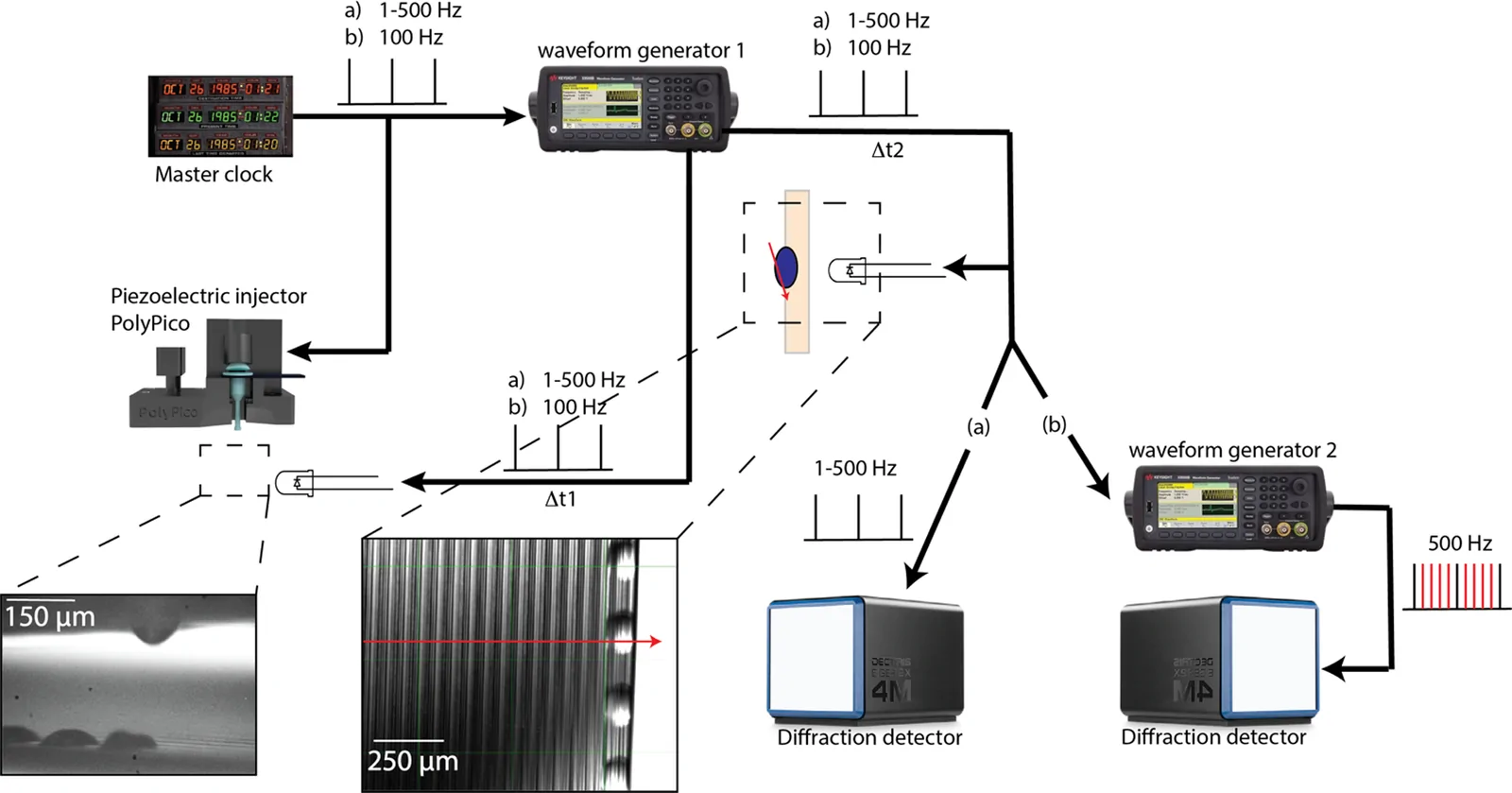
Triggering scheme of the tape drive and detector synchronization. (*a*) Because synchrotron X-ray pulses are essentially continuous on the time scale of the droplet motion, a master clock is defined by a signal generator which delivers TTL pulses at the desired frequency (*e.g.* up to 500 Hz). This master TTL signal triggers the sample droplet ejection by the PEI. The master clock also simultaneously triggers ‘waveform generator 1’ which will produce two TTL signals at the same frequency with delay Δ*t*1 and Δ*t*2, respectively. The first TTL signal triggers the strobed LED attached to the PEI; adjusting Δ*t*1 enables the visualization and calibration of the droplet ejection onto the moving Kapton tape. The second TTL signal triggers both the strobe LED behind the tape that illuminates the X-ray–droplet interaction region and the diffraction detector. By adjusting Δ*t*2, the droplet position on the tape is aligned to the X-ray beam (red arrow), thereby also triggering the detector to acquire diffraction data when droplets overlap the X-ray path. (*b*) Same as panel (*a*) except that the master clock runs at 100 Hz, defining the droplet ejection at 100 Hz. Another ‘waveform generator 2’ is placed before the detector generating a 500 Hz signal. This allows ejection of droplets at 100 Hz (black lines) while acquiring images at 500 Hz (red lines).

Experiments on XFEL beamlines typically rely on the pulse generated by the source as their master clock for synchronization. For our synchrotron experiments a local waveform generator (33600A, Keysight) created a ‘master’ TTL (transistor–transistor logic) pulse at the desired frequency up to 500 Hz [Fig. 2 ▸(*a*)] with which all other components were synchronized. This master signal was used to trigger the PEI sample ejection onto the tape, while a delayed signal was used to strobe an LED (4 V, white, 5 mm) to monitor the contact of the droplet with the tape. The droplets travelling on the tape to the X-ray interaction region were visualized using an on-axis viewing camera and an orthogonal camera. Only the orthogonal camera was illuminated by a strobed LED, positioned downstream behind the Kapton tape and offset to prevent shadowing of the diffraction images [Fig. 1 ▸(*c*)]. The strobed LED illuminated the interaction region and enabled us to synchronize the droplet arrival at the interaction region with the TTL signal by adjusting the LED delay and strobe pulse width. This adjusted TTL signal was used to trigger detector data collection. This ensured that detector readout was started just before the drop met the beam and ended just after it had passed through the interaction zone. Minimizing the exposure time without a droplet in the beam will allow us to maximize the signal-to-noise ratio and adjust the trigger point to correct for any slight alterations in ejection and tape movement. Future iterations of the device will use this technique to control a second ejection point where compounds will be mixed with the droplets on the moving tape, thus initiating chemical mixing, but this was not tested during the experiment described here.

### 2.3. Data collection

For data collection the linear tape speed (generated by the brushless rotating motor) was 25 mm s^−1^ (Table 1 ▸). The PEI dispensed droplets containing CTX-M-15 microcrystals at a frequency of 100 Hz, enabling the acquisition of a full data set of >20000 indexed images in about 30 min. The ejected droplet volume depends on several parameters, such as the PEI cartridge aperture and the amplitude and width of the signal controlling the ejection. The flying droplet velocity and volume were changed by increasing both the amplitude and width of the signal. In normal operation the droplet velocity before touching the tape was about 1–2 m s^−1^. We used a PEI aperture of 100 µm to avoid clogging the cartridge with the microcrystal slurry. The amplitude (20 to 60%) and width (50%) of the ejection signal were adjusted to deliver a droplet with a volume less than 500 pl. The droplet volume was estimated using the side-view camera (Fig. 2 ▸), which shows an average diameter of the droplet on the tape of 125 µm (160 µm vertically and 90 µm horizontally, due to tooling marks on the tape). The volume of the droplet on the tape is estimated using the hemisphere volume equation (1),
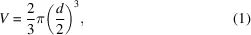
with *V* the hemisphere volume and *d* the droplet diameter on the tape.

To ensure the success of the experiment with a sub-optimal setup (no relative humidity control) we did not try to collect data with smaller droplets (<150 µm). While the droplets were dispensed at 100 Hz the detector collection rate was 500 Hz, which corresponds to a maximum of 2 ms exposure time [Fig. 2 ▸(*b*)]. The Eiger2X 4M detector was positioned 230 mm from the sample position, allowing a resolution of 2.4 Å in the inscribed circle and 1.72 Å in the corner of the detector. The 16 keV X-ray beam was focused to 10 × 10 µm with an estimated flux of 2 × 10^13^ photons s^−1^.

### 2.4. SSX data processing

CTX-M-15 serial synchrotron crystallography data were processed using *xia2.ssx* (Beilsten-Edmands *et al.*, 2024 ▸) (Table 2 ▸). Data underwent spotfinding, indexing and integration using *xia2.ssx*, while scaling and merging were carried out using *xia2.ssx_reduce*. The additional scaling parameter weighting.error_model.basic.min_Ih = 300 was required to improve error modelling due to varied intensity distributions. A minor additional unit-cell population was removed using unit-cell clustering via the parameter clustering.threshold = 5. Analysis of the indexed data versus frames collected suggested an approximate indexed hit rate of 10%. The structure was solved using PDB 7bh6 as the initial model. Structures were completed through rounds of iterative refinement in *phenix.refine* (Adams *et al.*, 2010 ▸) and manual model building in *Coot* (Emsley & Cowtan, 2004 ▸), with validation by *Molprobity* (Chen *et al.*, 2010 ▸) and *Phenix*.

The infrastructure used for serial crystallography at Diamond is identical to that employed for cryogenic and room-temperature rotation data collection. Consequently, the associated requirements for data processing and storage are already well established.

### 2.5. X-ray emission spectroscopy instrumentation on VMXi

**Figure 3:**
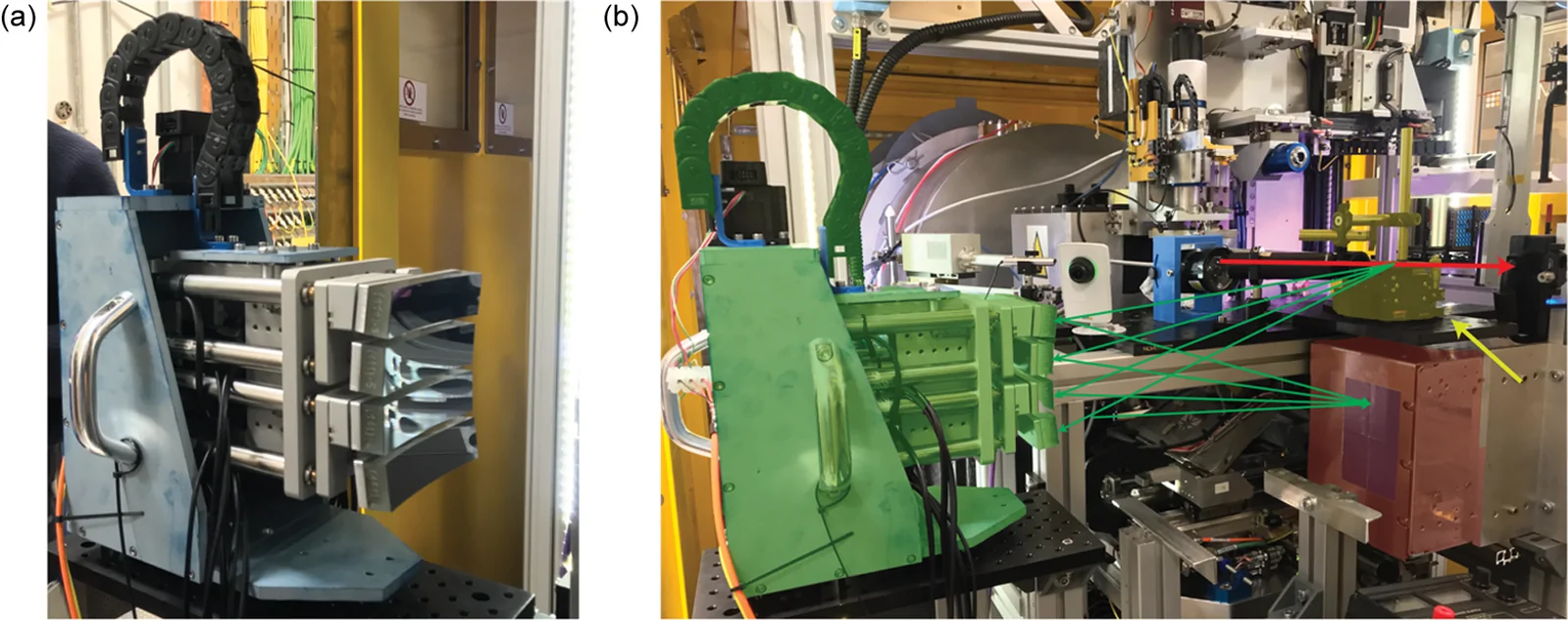
von Hamos XES spectrometer prototype installation on VMXi for Cu *K*α_1,2_ measurements. (*a*) A 4×1 bank of Si(111) analyser crystals with a 400 mm focal radius used to measure Cu *K*α_1,2_ XES data with the Si(444) reflection. (*b*) The setup without the helium cone and lead shielding. The XES detector (Tristan 1M) is highlighted in red, the analyser crystals bank is highlighted in green and the sample delivery system is highlighted in yellow (yellow arrow). The X-ray beam is indicated by the red arrow and the emitted photon path is shown with green arrows.

A von Hamos spectrometer to measure XES was developed in house at Diamond following similar principles to previously published designs (Fuller *et al.*, 2017 ▸) and installed on beamline VMXi. This consisted of three components: a crystal analyser array, a custom-built helium cone and a 2D X-ray sensitive detector (Fig. 3 ▸). The geometry was chosen to capture the emitted fluorescence orthogonal to the main beam path and diffracted down onto a detector below the sample position. The crystal bending radius was 400 mm, balancing the required spectral resolution with capturing a large solid angle within the available space, and minimizing clashes with existing equipment. Unlike scanning spectrometers, the von Hamos geometry only focuses the X-rays horizontally, so the Bragg angle and associated energy vary across the vertical face of the analyser crystal. Provided the analyser crystal has sufficient height, the entire spectrum can be collected simultaneously (Fig. 3 ▸), producing a vertical signal on the detector.

For the proof-of-concept experiments described here we used analyser crystals arranged in a vertical block. Both Cu *K*α_1,2_ and Fe *K*α_1,2_ emission lines were collected using four Si(111) (reflection 444) or two Ge(110) (reflection 440) crystals, respectively. The Si(111) and Ge(110) crystals had bending radii of 400 mm and 250 mm, respectively. Iron measurements used analyser crystals kindly borrowed from XRSTech. Each analyser crystal could be independently adjusted in focal length, pitch and yaw, which was used to overlap the separate signals to maximize the signal-to-noise ratio. This adjustment was carried out by eye using reference metal foils or chemical solutions. For future designs we aim to incorporate automated calibration routines to ensure a consistent setup.

A simple temporary helium cone was constructed from transparent acrylic with epoxy joints. X-ray input and output windows were lined with Kapton to minimize X-ray absorption. The cone was mounted between the analyser crystals and the detector and maintained at a slight positive pressure. The cone reduced the air absorption path to only 10 mm, improving the signal-to-noise ratio (Fig. S3). Further significant improvements to the helium cone for ease of mounting and minimal helium usage are currently under investigation. The XES detector was extensively masked with lead tape (5 mm thick) to avoid contamination by X-rays directly scattered from the equipment on the beamline near the inter­action region.

The X-ray detectors used were a Merlin 0.25M (Plackett *et al.*, 2013 ▸) and a Tristan 1M (Omar *et al.*, 2022 ▸) Timepix3 (Poikela *et al.*, 2014 ▸) for the Fe and Cu signals, respectively. Both detectors were provided by the Diamond detector group for testing within our overall experimental constraints and they fitted within the limited space on VMXi. They both had a 55 µm vertical pixel pitch, causing minimal degradation to spectral resolution. The Tristan detector is intended to be implemented for future applications and has a 113.8 × 28.3 mm active area, which has sufficient vertical height to measure multiple different emission lines simultaneously. The Merlin was tested for the initial measurements because its overall form factor was more compact, and included detector drivers that were already integrated with the data collection software. We anticipated no fundamental difference with photon detection because both the Merlin and Tristan are based on the same underlying Medipix technology. However, the Tristan 1M detector exploits the Timepix3 ASIC and can work in event-driven mode in addition to the standard frame-based mode. In event mode, the Timepix3 time stamps each event at each pixel with an accuracy of 1.5625 ns, which thereby provides additional capabilities to analyse time-resolved XES data, potentially for the strongest signals on an orbit-by-orbit basis of the electron bunches around the Diamond storage ring. The same downstream interaction point was used as for the SSX measurements, with the extended on-axis viewing system and re-focused beam, but it required a different supporting structure to mount the von Hamos crystal array and Tristan detector.

### 2.6. Sample preparation for XES

Iron- and copper-containing salts [Cu^I^Cl (229628, Merck), Cu^II^Cl_2_ (222011, Merk), K_3_Fe(CN)_6_ (10266370, Fisher Scientific) and K_4_Fe(CN)_6_ (11452378, Fisher Scientific)] were used as solids mounted between O_2_-tight foils using standard chip-less chips (Doak *et al.*, 2018 ▸). Iron foil used as a standard was purchased from Advent Research Materials Limited (FE163808).

*Ac*NiR was purified and microcrystals prepared as previously described (Horrell *et al.*, 2016 ▸); their size and density were assessed by visible-light microscopy. Crystals of *Ac*NiR were tested for diffraction quality on the I24 beamline (Diamond) before transfer into a high-viscosity medium for extrusion. Two parts of *Ac*NiR microcrystal slurry were mixed with three parts of monoolein (HR2-435, Hampton Research) to form the lipidic cubic phase (LCP) prior to loading in the high-viscosity extruder.

### 2.7. Data collection for XES

For calibration standards, simple mountings were used to position the different solid samples at the focal point. Metal foils held using kinematic posts were oriented at 45° to the X-ray beam to ensure a small source point to improve spectral resolution, while also providing an escape path for the emitted photons onto the 2D Tristan X-ray detector. The *K*α_1,2_ XES of the iron foil was collected with 60 s of data acquisition, and data for the iron and copper salts were collected with 6 s of data acquisition. Protein microcrystals were ejected into the interaction point using a high-viscosity extruder and ‘catcher’ (Weierstall *et al.*, 2014 ▸). This delivery system was used due to its simplicity to install and align, albeit with higher background and not permitting synchronization of data collection.

The LCP was extruded at a rate of 50 to 150 nl per minute through a nozzle size of 75 µm inner diameter that extrudes a similar diameter column of high-viscosity media. The flow rate of the ejector was varied to ensure a stable flow, resulting in an absorbed dose range of 12–35 kGy depending on the flow rate. X-ray diffraction data (not shown) were measured simultaneously with the XES data. In order to generate individual frames for the XES data the Tristan detector was sent the associated TTL pulse for the X-ray detector readout. This could be used to turn the continuous readout data into a stack of associated images. *K*α_1,2_ XES data from *Ac*NiR microcrystals were collected with an accumulated acquisition time of 2400 s.

### 2.8. Data processing for XES

XES data recorded either on the Merlin 0.25M or on the Tristan 1M (based on the Timepix3 chip) detector were processed using in-house developed software. Data reduction and processing followed procedures as outlined previously (Fransson *et al.*, 2018 ▸). Data collected from the Merlin detector could be summed and reduced directly. Tristan data were processed into associated readout frames utilizing the associated TTL pulse from the Eiger detector using software developed at Diamond. Tristan XES data were converted into a set of images (Hatcher *et al.*, 2022 ▸) that matched the X-ray diffraction frame number, or 30 images for foil/salt data. Hot pixels were removed with a given intensity threshold. A region of interest (ROI) around the spectrum was selected and pixel intensities along the *y* axis (vertical) were summed to produce the raw spectrum. Pixel intensities in two thin bands beyond and below the ROI were also vertically summed as background and subtracted from the raw spectrum. The intensity of the background-subtracted spectra was normalized to the maximum intensity of the *K*α_1_ peak. In order to calibrate the XES data, Fe *K*α_1,2_ spectra were measured from an Fe foil (Fig. S4) and the energy was calibrated for each vertical pixel by interpolating to a known *K*α_1,2_ emission line (Thompson *et al.*, 2009 ▸). For the Cu data, CuCl_2_ data were measured using the experimental set up reported here, and these data were energy calibrated using CuCl_2_ data collected on I20-Scanning at Diamond Light Source by Dr Sherwin (University of Oxford).

## 3. Results

### 3.1. Tape drive operation and delivery of droplets

We established that a moving closed-loop belt of 6 mm wide Kapton, similar to that used previously in the DoT LCLS system, was the best performing option. However, because we were using much smaller droplets, we needed a more precise mechanical performance, very tight motion control and highly precise ejection. Achieving the required performance necessitated the development and testing of several iterations of the tape drive system and its associated wash–dry mechanism. The version used in the present study represents an intermediate design – less precise than the final design, yet sufficiently robust to enable proof-of-concept experiments on VMXi.

**Figure 4:**
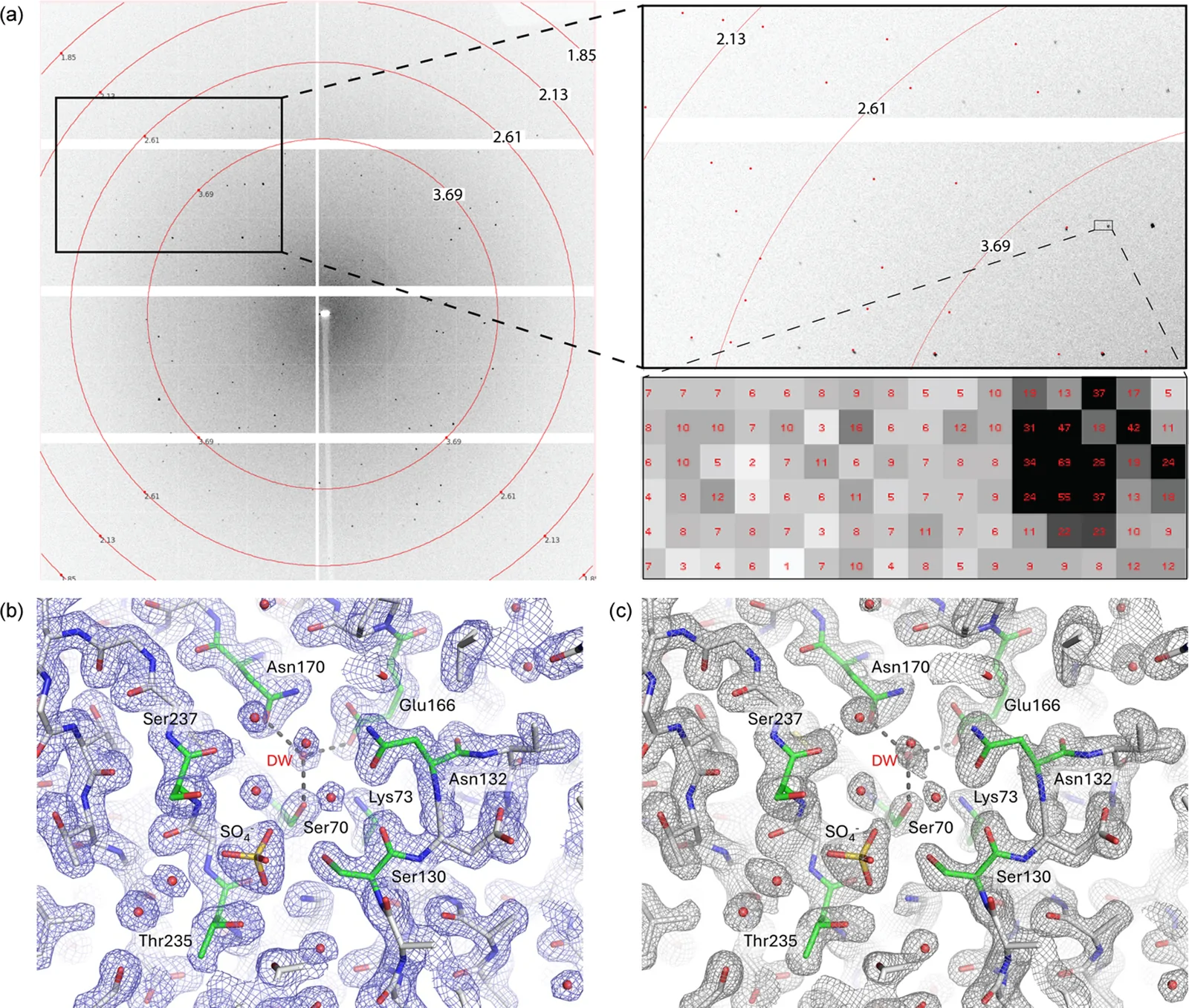
Serial synchrotron crystallography structure of serine β-lactamase CTX-M-15, originally found in *Klebsiella pneumoniae* IS53, determined using our tape drive system on VMXi. (*a*) Diffraction pattern measured from ∼8 µm microcrystals within ∼500 pl droplets on a tape moving at 0.25 m s^−1^ with 2 ms total exposure time, during which crystals were in the 16 keV X-ray beam (10 × 10 µm, pink beam) for a maximum of ∼720 µs. (Top inset) Expanded regions of the detector image with an example spot profile for a ∼2.1 Å reflection and (bottom inset) an indication of the background levels, showing the pixel intensity across the region where the tape scattering may be observed. (*b*) The 2*F*_o_ − *F*_c_ electron-density map at 1.83 Å resolution shown as a blue mesh contoured at 1.2σ. The carbon reading atoms of key active site residues are coloured green and labelled. Sulfate (labelled) from the crystallization condition is bound at the active site; the catalytic deacyl­ating water is labelled DW and hydrogen-bonded to Ser70, Glu166 and Asn170. The figure was created in *PyMol* (https://www.pymol.org/). (*c*) 2*mF*_o_ − *DF*_c_ composite omit map with 5000 K Cartesian simulated annealing (created in *Phenix* with 5% of atoms omitted at each step) of the 1.83 Å resolution CTX-M-15 data, shown as a grey mesh contoured at 1.2σ.

First, the capability to position droplets consistently on the moving tape and synchronize this with X-ray exposures was tested. X-ray diffraction data were collected to 1.83 Å resolution from microcrystals of the CTX-M-15 extended-spectrum β-lactamase within droplets on the tape, using a linear tape speed of 25 mm s^−1^. When the PEI was positioned 60 mm from the interaction region, it took 2.4 s for the microcrystal-containing droplet to reach the X-ray beam. In this configuration, the resulting diffraction patterns were of good quality and were successfully processed [Fig. 4 ▸(*a*)]. However, when the PEI was placed more than 100 mm away, no diffraction patterns were observed, probably due to dehydration effects under the relative humidity of 20% maintained in the VMXi hutch. Consequently, the final design will include an environmental chamber that can adjust humidity levels and, when necessary, also eliminate O_2_ to better than 100 p.p.m., according to our preliminary test on the tape drive enclosure.

To ensure that the X-ray beam consistently intersected with the droplets on the tape, we implemented a data collection strategy in which droplets were ejected at 100 Hz, while the detector acquired frames at 500 Hz with a 2 ms exposure time [Fig. 2 ▸(*b*)]. Immediate feedback was provided by monitoring the acquired image size. When a droplet was hit by the X-ray beam the resulting image size was larger, due to the solvent ring scattering, than an image when no droplet was present in the X-ray path. We used this tool to move the tape with a motorized translation stage as close as possible to the X-ray beam until we observed a pattern corresponding to a larger image in one of every five collected (Fig. S5). By combining this approach with a Kapton tape positioned slightly off axis relative to the X-ray beam, we ensured that the beam grazed the tape surface, with even larger images being observed when the beam intersects with the tape. This geometry minimized tape-induced background scatter in the diffraction data (Fig. S6), unlike the configuration used by the DoT which requires tape background correction tools (Fuller *et al.*, 2017 ▸).

The main advantage of using the PEI for ejection of droplet-containing microcrystal slurry is to reduce sample consumption. Indeed, the PEI is capable of ejecting ∼150 pl droplets or less, while the ADE method (Fuller *et al.*, 2017 ▸; Hadimioglu *et al.*, 2016 ▸) produces droplets in the range of 2 to 5 nl, depending on the transducer frequency and ejection parameters. The droplet size generated by the PEI depends on the cartridge aperture. For CTX-M-15 we used a 100 µm aperture to avoid clogging at the tip of the cartridge, and to compensate for the lack of relative humidity control we used higher ejection parameters than usual to create droplet volumes around 500 pl and 125 µm diameter on the tape. Data collection took 32 min, which means that with a droplet ejection frequency of 100 Hz, we used about 100 µl of microcrystal slurry (with a density of 10^8^ crystals ml^−1^) for a high-quality data set. This sample consumption is comparable with the fixed-target approach (Horrell *et al.*, 2021 ▸; Jaho *et al.*, 2024 ▸). The comparison with the ADE DoT tape drive for the same sample (CTX-M-15 at the same density) (Butryn *et al.*, 2021 ▸) shows that the prototype tape drive with PolyPico is four times more efficient (17130 lattices collected per milligram of protein versus 4394 for ADE) (Table S1).

During the 32 min data collection, 192000 droplets of ∼500 pl volume containing CTX-M-15 microcrystals were ejected, with an average of 50 crystals in each droplet for a crystal density of 10^8^ crystals ml^−1^. With a 10% hit rate we indexed 20556 lattices (Table S2). In total we dispensed about 9600000 crystals, corresponding to 1.2 mg of protein.

The SSX data produced a clearly interpretable electron-density map to 1.83 Å resolution [Figs. 4 ▸(*b*) and 4 ▸(*c*)], yielding a refined structure (Table 2 ▸) from an estimated maximum exposure time of 720 µs (12.5 kGy) per crystal and 2 ms total X-ray time per image. With an optimized setup we anticipate reducing the sample consumption by at least a third and electronically gating the detector to match better the time taken for each drop to traverse the X-ray path, with a commensurate reduction in noise.

### 3.2. XES data

Prototype experiments for X-ray emission spectroscopy had two principal aims, (i) ensuring that anticipated *K*α_1,2_ spectral changes due to different oxidation states were detectable given the experimental geometry and spectral resolution, and (ii) ensuring a detectable signal from protein microcrystals given the limited metal concentration within the crystal structure. To assess difference features, the transition metals iron and copper were used as common catalytically active metals in metalloenzymes. Iron salts potassium ferricyanide K_3_Fe(CN)_6_ and potassium hexa­cyano­ferrate K_4_Fe(CN)_6_ were chosen to produce Fe^3+^ and Fe^2+^ signals, respectively. For copper, copper(II) (Cu^II^Cl_2_) and copper(I) (Cu^I^Cl) chlorides were selected to produce Cu^2+^ and Cu^1+^ signals. In order to assess whether sufficient signal was measurable from protein microcrystals, the metalloenzyme *Ac*NiR was used as a model system. *Ac*NiR is easily purifiable, has a well characterized micro-crystallization condition and contains two catalytically important copper sites for which catalysis can be driven by a reduction in the X-ray beam, which has previously been well characterized (Horrell *et al.*, 2016 ▸).

**Figure 5:**
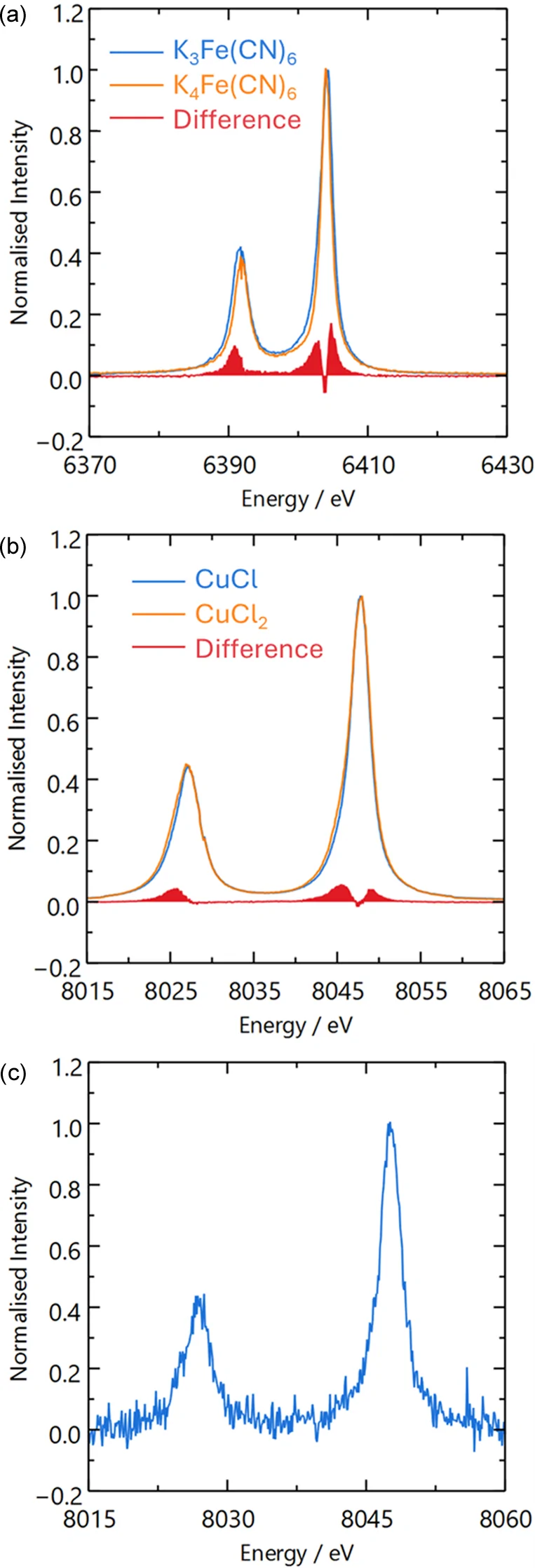
XES spectra. (*a*) *K*α_1,2_ XES of Fe salt powder grains [K_3_Fe^III^(CN)_6_ and K_4_Fe^II^(CN)_6_] and difference spectrum, K_3_Fe^III^(CN)_6_ − K_4_Fe^II^(CN)_6_. (*b*) *K*α_1,2_ XES of Cu salts (Cu^I^Cl and Cu^II^Cl_2_) and difference spectrum, Cu^II^Cl_2_ − Cu^I^Cl. (*c*) *K*α_1,2_ XES of *Ac*NiR microcrystals from the two mononuclear Cu^II^ metal centres.

Iron spectra were measured from an iron foil and the ferrous salts were recorded on a 0.25M Merlin detector using two Ge(110) crystals at 250 mm working distance. Energy calibration was carried out against previously published Fe spectra (Lafuerza *et al.*, 2020 ▸). The Fe spectra, as anticipated, produced a strong signal and a clear difference spectrum. These were in agreement with previously reported spectra for these compounds. Copper spectra were measured with a 1M Tristan detector replacing the Merlin and four Si(111) crystals at 400 mm bending radius. The spectra of the Cu^II^Cl_2_ and Cu^I^Cl salts showed a good signal-to-noise ratio and a clear difference spectrum [Fig. 5 ▸(*b*)]. These results demonstrate that the setup can measure Fe and Cu *K*α_1,2_ spectra with a difference spectrum associated with the change in oxidation state.

Finally, we measured XES spectra from protein microcrystals using the same setup as for copper salts. Crystals of *Ac*NiR (Horrell *et al.*, 2016 ▸) were delivered into the X-ray beam in an LCP medium using a high-viscosity extruder. The extruder is a well characterized and frequently used sample delivery method in serial crystallography, allowing us to focus on optimizing the XES experiment, although a higher X-ray background is anticipated than for the tape drive droplets. A clearly discernible *K*α_1,2_ doublet was recorded [Fig. 5 ▸(*c*)] as an average signal collected over 2400 s that concomitantly produced a reasonable hit rate for the forward diffraction (data not shown). Comparing the calibrated spectrum centre of mass (COM) with the previously collected salt spectrum did not allow assignment of the copper oxidation state in the microcrystals. Copper nitrite reductase contains two copper ions per 37 kDa monomer of the functional trimer. These copper ions have different functions and active site ligation which may lead to differences in their rate of X-ray reduction, with the resulting XES spectra likely to be an average of the signals from both copper environments and potentially also a mixed oxidation state in one or both of these.

## 4. Discussion

### 4.1. Main outcomes so far

Our proof-of-principle experiments yielded two main outcomes, (i) demonstrating that collection of good quality diffraction data on a synchrotron beamline is very much possible from microcrystals dispensed onto a moving tape using ∼500 pl droplets, and (ii) Cu *K*α XES spectra from enzyme microcrystals delivered through the beam using a high-viscosity extruder could be measured using the von Hamos geometry. In both cases, and despite the ongoing project being in a developmental stage with non-optimized setups, good quality data were obtained for both diffraction and XES. Using the prototype tape drive setup described here, a crystal structure was determined from protein microcrystals using about 100 µl of microcrystal suspension. Diffraction data for CTX-M-15 were collected in about 30 min, producing 20556 indexed diffraction images to 1.83 Å resolution. The resulting refined SSX structure was similar to those obtained from experiments at SACLA (DoT, PDB 7bh3), PAL-XFEL (fixed target, PDB 9to1) and Diamond I24 (fixed target, PDB 9to5), and to a single-crystal structure determined at 100 K at a synchrotron (PDB 4hbt). The major difference between these structures is a slight movement of one of the key catalytic residues – Lys73 (particularly at room temperature versus 100 K) – and of the bound water molecule responsible for the breakdown of the covalent β-lactam acyl­enzmye complex; otherwise, the overall structures are similar, with Cα RMSDs against the VMXi structure (calculated using the *PDBeFold* protein structure comparison service at the European Bioinformatics Institute, https://www.ebi.ac.uk/msd-srv/ssm) (Krissinel & Hendrick, 2004 ▸; Krissinel & Hendrick, 2005 ▸) of 0.1 (SACLA, DoT, room temperature), 0.14 (PAL-XFEL, fixed target, room temperature), 0.18 (I24, fixed target, room temperature) and 0.2 (100 K structure).

For this proof-of-concept work we focused on creating a simplified tape drive mechanism to confirm that it was possible to dispense sub-nanolitre droplets of sample onto a moving tape, then present them to the X-ray beam and record diffraction data on a synchrotron beamline with sub-microsecond X-ray exposure. On VMXi at Diamond the challenge of matching/synchronizing the sample to the beam is slightly different to that at XFEL sources. The synchrotron beam is not pulsed (hence guaranteeing the presence of X-rays at the time the droplet crosses the interaction point). Moreover, the total dose per droplet is lower and dependent on the tape velocity, which makes synchronization a bit more difficult. For instance, at XFELs we typically observe droplet explosions and the XFEL X-ray pulse is driven by a master clock and synchronized with the detectors, so the challenge is limited to synchronizing the droplet delivery to the X-ray pulse by adjusting the droplet ejection delay time relative to the master clock. Thus, synchronization can be achieved by visual observation of droplet destruction and by observing the X-ray scattering from the droplets in the forward detector images. At synchrotrons it is not as ‘simple’ as that. Unlike the XFEL pulsed X-ray beam, the synchrotron X-ray beam can be considered to be constant. Therefore, to reduce unwanted background scattering caused by the constant beam, the detector and the droplet delivery into the interaction region must be synchronized. If this is not done correctly, the data are significantly limited by the ratio of the weaker signal and the higher noise. Consequently, we used X-ray scattering from the water in the droplets that correlates with detector image file size to optimize synchronization and maximize signal-to-noise ratio.

The results with the prototype XES system are also very promising. In this proof-of-principle experiment we used a reduced solid angle (only up to two or four von Hamos analyser crystals out of the planned 16 in the final design). Furthermore, the sample delivery available at the time, the LCP extruder, was far from ideal, since it can be difficult to control with constant flow and produces a large X-ray background leading to a much weaker signal-to-noise ratio. Energy calibration of the Fe and Cu *K*α_1,2_ XES spectra using the methods reported in Section 2.8 gave an energy axis with a pixel-to-pixel resolution of 0.1 eV, which we anticipate being adequate for tracking changes to spin state and oxidation state across a time-resolved series of XES measurements, as these changes typically incur energy shifts of the order of 0.1–2 eV. The *K*α_1,2_ XES spectra for the iron salts [K_3_Fe(CN)_6_ and K_4_Fe(CN)_6_] are consistent with literature values for both the *K*α_1_ FWHM and the COM (Lafuerza *et al.*, 2020 ▸) (Table 3 ▸).

Successful measurements of both Fe and Cu *K*α_1,2_ XES demonstrate the versatility of the von Hamos spectrometer for element-specific X-ray spectroscopic measurements by interchanging suitable analyser crystals. As expected, the Cu *K*α_1,2_ XES spectrum for *Ac*NiR has a lower signal-to-noise ratio (Table S3) when compared with the salt spectra; however, by calculating the *K*α_1_ FWHM and COM we anticipate we will be able to track changes in the oxidation state across a time-resolved reaction series. Ambiguity in assigning an oxidation state to the measured *Ac*NiR sample arises from the two structurally distinct mononuclear copper sites. The XES signal obtained is an average of the two sites within *Ac*NiR; in order to overcome this in the comprehensive commissioning tests, we will measure standards (salts, enzymes *etc.*) in known oxidation states for comparison.

To obtain the copper enzyme *K*α_1,2_ XES spectra shown (Fig. 5 ▸), we averaged data over a period of 2400 s (40 min), which is comparable with the total time required to obtain a typical serial crystallographic diffraction data set from either a tape drive, several fixed targets or viscous-media extruder strategies. In a typical SSX data set, approximately 10000 still images are merged wherein the X-ray exposure time per crystal is a function of the sample delivery strategy and beamline parameters, generally ranging from ∼10 µs at diffraction-limited sources to ∼2–10 ms per still image on beamlines with Pilatus or Eiger detectors, or 500 µs using a Jungfrau detector. With the improvements to the von Hamos spectrometer system that we are undertaking (many more analyser crystals, lower background from the sample medium, only accumulating signal that is correlated with diffraction) we anticipate the time required to obtain such X-ray spectroscopic data will decrease significantly.

### 4.2. Planned improvements and a combined droplet-on-demand and XES system

While the results we have obtained from these prototype systems are promising, they represent the early steps on the development pathway to a much more capable system. We expect the final design to deliver much greater control and sensitivity, making such experiments more routine and providing a useful capability to the time-resolved structural biology community.

We will use coordinated trigger signals and electronic gating of the X-ray diffraction detector to measure diffraction data only when a droplet is traversing the beam, greatly improving the signal-to-noise ratio and allowing for shorter exposure times. It is nevertheless notable that in this study we obtained excellent quality diffraction data even without this improvement. The final system with an environmental chamber will also improve humidity control (to avoid dehydration of samples) and enable implementation of controlled anaerobic conditions to permit data collection from oxygen-sensitive microcrystals, including many metalloenzymes with Fe, Cu, Mn and/or Ni catalytic centres. As demonstrated here, reducing the droplet size from 2 to 5 nl (ADE) to between 50 and 500 pl (PEI) greatly reduces the quantity of sample required to measure a data set, increases the signal-to-noise ratio and shortens the mixing times when executing a drop-on-drop experiment. The constant X-ray source of the synchrotron compared with the 10–50 fs pulse duration at an XFEL is also beneficial for reducing the quantity of sample needed, as passing droplets through a beam that is always on provides more opportunities for crystal interactions with the beam that may yield data.

The final version of the von Hamos spectrometer aims to measure XES from ∼150 pl droplets or smaller and record the data using the newly developed Tristan 2M event-counting detector. The final crystal array is planned to comprise four sets of four analyser crystals (110 × 25 mm) with a 400 mm radius, positioned to diffract back and focus the emitted fluorescence photons into an energy-dispersive line on the 2D detector. The use of 16 analyser crystals, compared with the four used in the prototype setup, is expected to measure four times the XES signal intensity, which will result in a better signal-to-noise ratio. The analyser crystals are placed orthogonal to the beam path and aligned such that, on the vertical plane, the lattice spacing creates a discrete diffraction point on the detector depending on the energy. By selecting analyser crystals with different lattice parameters, the device is planned to be able to be used to observe up to four separate *K*α_1,2_ emission lines (initially Fe, Cu, Mn and Ni) or, if all 16 crystals are used, we aim to be able to analyse *K*β spectra for the same elements. The strength of the XES signal will also be dependent upon the sample, for example the size of the microcrystals and the concentration of the metal ion in the sample, which will vary from system to system based on the number of metal ions in the protein, as well as on the size and the packing density of the protein.

The signal-to-noise ratio for protein *K*α_1,2_ XES spectra is expected to improve with the planned advances to the sample delivery system and von Hamos spectrometer. Moreover, the time-stamp capability and nanosecond temporal resolution for each pixel in the Tristan 2M detector provide opportunities to measure XES data from intensely emitting samples on an orbit-by-orbit basis of the electron beam circulating in the storage ring. This enables much better precision in both the timing and location of the XES signal (1.5625 ns per pixel) compared with the current Eiger detector (500 Hz, 2 ms per image frame) used in the forward direction for diffraction data. The Tristan 2M XES detector will also provide strategies to synchronize better the overlap of picolitre-volume droplets traversing the X-ray beam on VMXi and thereby improve the signal-to-noise ratio of the XES signal. We can also easily envision a future configuration where a Tristan 10M is used for diffraction that is synchronized with the Tristan 2M for X-ray spectroscopy.

Serial data collection requires significant computational resources for data analysis in close to real time. We note that effective automated processing of serial crystallographic data within *xia2.ssx* is in place at Diamond and used with both fixed-target and high-viscosity extruder sample delivery. While raw data volumes may be large, all data measured at Diamond Light Source are placed in long-term storage, and processed data files are no larger than for conventional macromolecular crystallography experiments. In the case of XES, data volumes are smaller. For successful combined experiments the ability rapidly to obtain electron-density maps correlated with X-ray spectroscopic data in order to guide the next experimental steps is highly desirable.

In summary, these proof-of-principle experiments demonstrate the feasibility of a synchrotron-based instrument, combining droplet-on-demand and XES, for routine easy-to-use time-resolved crystallography. The first time anyone does anything, it is a somewhat challenging and requires subsequent optimizations. Fortunately, the synchrotron environment allows for higher-frequency ‘test/improve’ iteration cycles.

The overall goal of our ongoing project is to create a routine and easy-to-use setup for time-resolved serial crystallography that is fully correlated with spectroscopic methods and with samples under controlled environmental conditions. Based upon our progress to date, we are certain it will deliver much improved functionality for the scientific community. Our droplet delivery strategy uses about a tenth of the sample required by previous methods, and the smaller droplets will also improve the temporal resolution for the ubiquitous systems that require ligand mixing. The full extent of the improvements in terms of use, functionality and data quality will be evaluated during our commissioning phase, the results of which will be the subject of additional reports.

The proof-of-concept experiments presented here – albeit comparatively cumbersome and executed during the project definition phase – clearly demonstrate feasibility and value. Some of the aspects that make these types of experiment difficult are currently still true for applications at XFELs that were initiated more than ten years ago. Some key advantages of the system we are developing include improved access due to the more readily available synchrotron beamtime. Moreover, because we are designing and building our system for portability to be used at multiple synchrotrons as well as at XFELs, the science drivers and user community will benefit from improved training that more efficiently uses precious sample and beamtime at large-scale facilities.

## Supplementary Material

Additional figures and tables. DOI: 10.1107/S2052252526005658/rs5009sup1.pdf

PDB reference: serine β-lactamase CTX-M-15, 9yrd

## Figures and Tables

**Table 1:** Crystal exposure time to X-rays versus tape speed

Linear tape speed (mm s^−1^)	Maximum time required for an 8 µm crystal to traverse a 10 µm X-ray beam (ms)	Dose† (kGy)
10	1.8	31.3
25	0.72	12.5
50	0.36	6.3
100	0.18	3.1
200	0.09	1.6
500	0.036	0.6
1000	0.018	0.3

† Dose calculated with *RADDOSE-3D* (Bury *et al.*, 2018 ▸; Paithankar & Garman, 2010 ▸) based on an 8 × 3 × 3 µm CTX-M-15 crystal and 100% transmission at 16 keV on VMXi with a flux of 2 × 10^13^ photons s^−1^ on VMXi.

**Table 2:** Diamond Light Source VMXi room-temperature crystallographic data collection and refinement statistics for CTX-M-15 crystals Data for the high-resolution shell are shown in parentheses.

Data collection	
Resolution range (Å)	58.72–1.83 (1.86–1.83)
Space group	*P*2_1_2_1_2_1_
Unit-cell edges *a*, *b*, *c* (Å)	44.01, 45.55, 117.44
No. crystal lattices merged	20556
Total reflections	2016541 (11570)
Unique reflections	21932 (1038)
Multiplicity	91.9 (11.1)
Completeness (%)	99.8 (98.2)
*I*/σ(*I*)	9.2 (0.8)
*R*_split_	0.117 (1.087)
CC_1/2_	0.966 (0.305)
	
Refinement	
Resolution (Å)	58.72–1.83
No. of reflections	21865
*R*_work_/*R*_free_ (%)	16.58/19.94
No. of non-H atoms	
Protein	1976
Solvent	205
*B* factors (Å^2^)	
Protein	17.32
Solvent	28.75
R.m.s. deviations	
Bond lengths (Å)	0.005
Bond angles (°)	0.77
Ramachandran (%)	
Outliers	0.39
Favoured	98.44
PDB code	9ryd

**Table 3:** XES data measured on VMXi using a von Hamos spectrometer and a Merlin 0.25M or Tristan 1M detector

Sample	Formal valence	Experimental *K*α_1_ FWHM (eV)	COM (eV)
K_3_Fe(CN)_6_	+3	2.5	6399.0
K_4_Fe(CN)_6_	+2	2.0	6398.4
CuCl	+1	2.8	8046.3
CuCl_2_	+2	3.1	8044.4
*Ac*NiR	+2	2.7	8042.0

## Data Availability

Coordinates and structure factors have been deposited in the Protein Data Bank with accession number 9ryd. XES spectra are available on request.
