# Supplementary material for: Droplet-on-demand tape drive and X-ray emission spectroscopy prototypes for time-resolved serial crystallography on VMXi at Diamond Light Source
Source: IUCrJ. 2026 Jul 30;13(Pt 5):635–49. doi: 10.1107/S2052252526005658 (PMC13548830; doi:10.1107/S2052252526005658)
Supplement: Supplementary file 1 [file m-13-00635-sup1.pdf]

IUCrJ

Volume 13 (2026)

Supporting information for article:

Droplet-on-demand tape drive and X-ray emission spectroscopy prototypes for time-resolved serial crystallography on VMXi at Diamond Light Source

Pierre Aller, Juan Sanchez-Weatherby, Abbey Telfer, Robert Bosman, Nicholas Devenish, Philip Hinchcliffe, Sam Horrell, Jophy Ip, Richard Littlewood, Andrew Male, Eva Gimenez Navarro, Urszula Neuman, Jos J. A. G. Kamps, David Omar, Laura Parkinson, Muthraj Pandi, Nico Rubies, James Sandy, Anastasiia Shilova, James Spencer, Jonathan Spiers, John P. Sutter, Amy J. Thompson, Catherine L. Tooke, Ben Williams, Tiankun Zhou, Michael A. Hough and Allen M. Orville

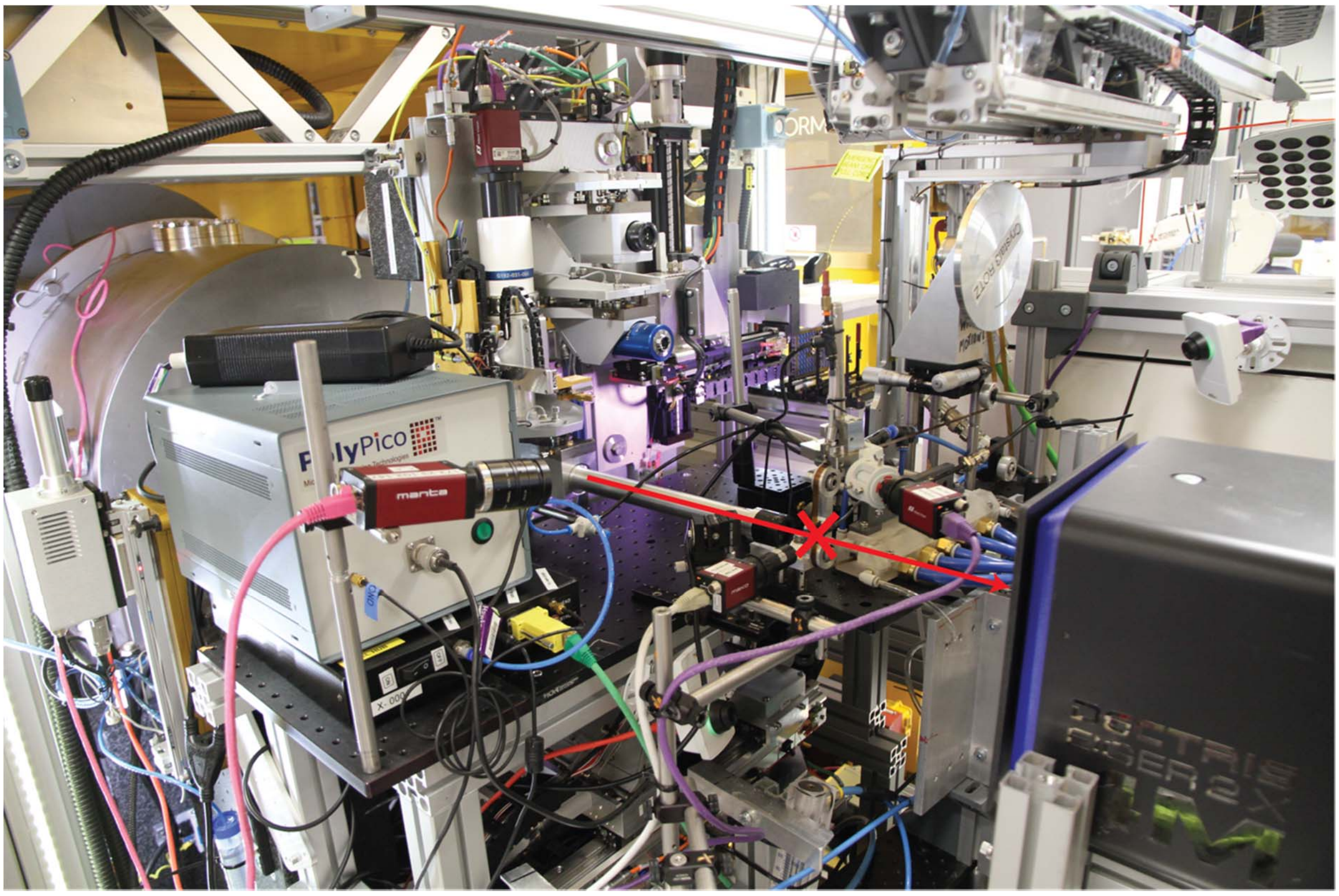

Figure S1 Tape drive setup on VMXi with the X-ray beam direction and the interaction region represented by a red arrow and a red cross, respectively.

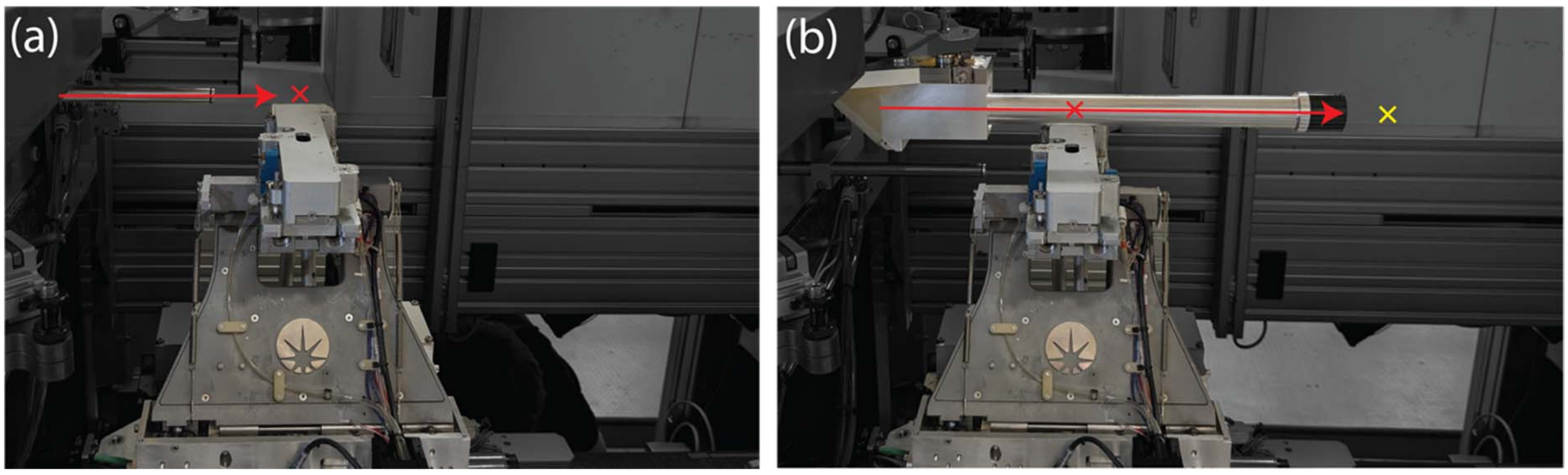

Figure S2 Interaction region with X-rays. (a) The X-rays are focused to the position of the red crosshair when VMXi is in *in situ* mode. (b) To leave enough space to install the tape drive the beam can be refocused 250 mm downstream (yellow crosshair). Adding an infinity-corrected objective lens allows the use of the existing VMXi beamline on-axis viewing system. The beam direction is shown by the red arrow.

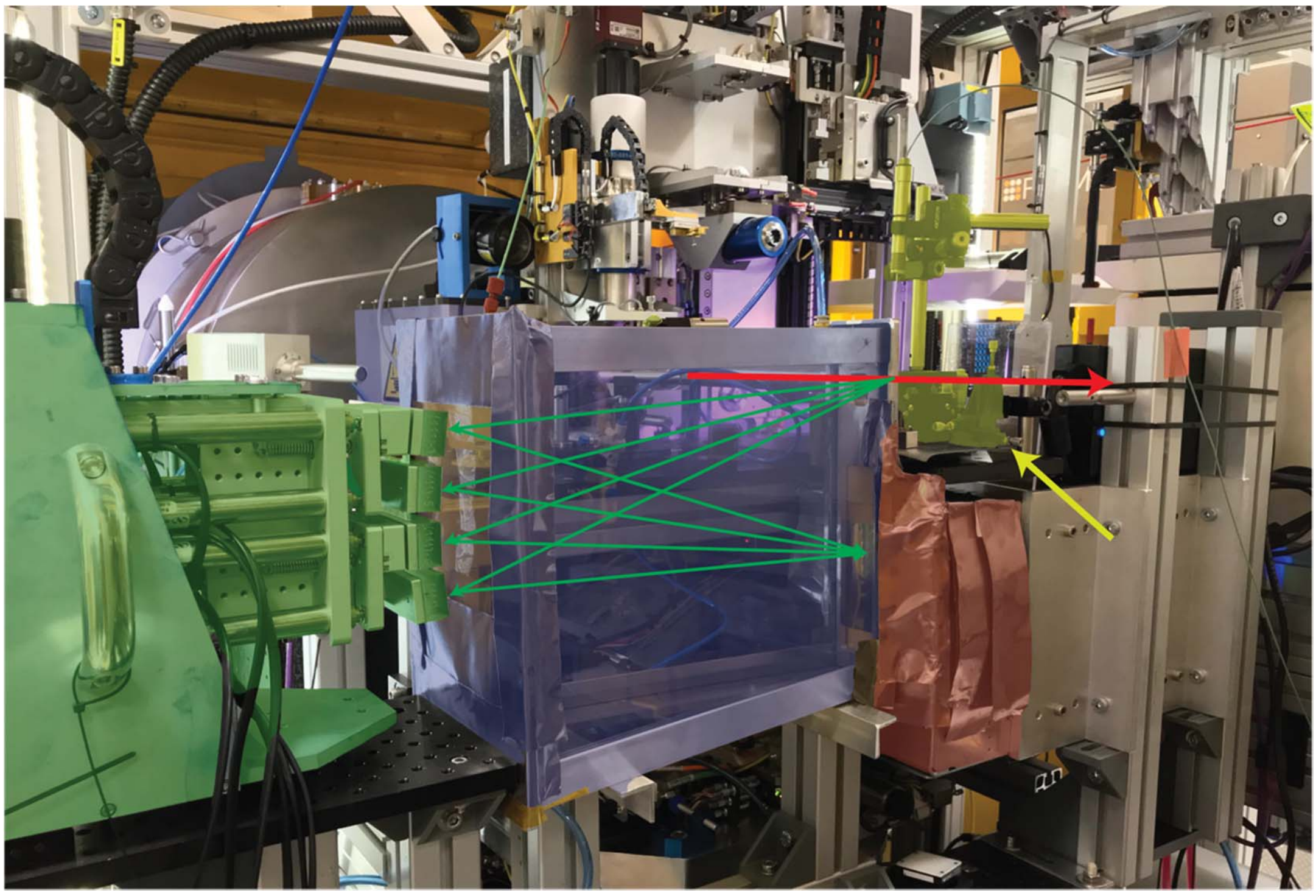

Figure S3 von Hamos XES spectrometer prototype installation on VMXi for Cu protein measurements. Setup shown with helium cone highlighted in blue and lead shielding in red shading around the XES detector (Tristan1M). The analyser crystals bank is highlighted in green and the sample delivery system is highlighted in yellow (yellow arrow). The X-ray beam is indicated by the red arrow. The emitted photon path shown with green arrows.

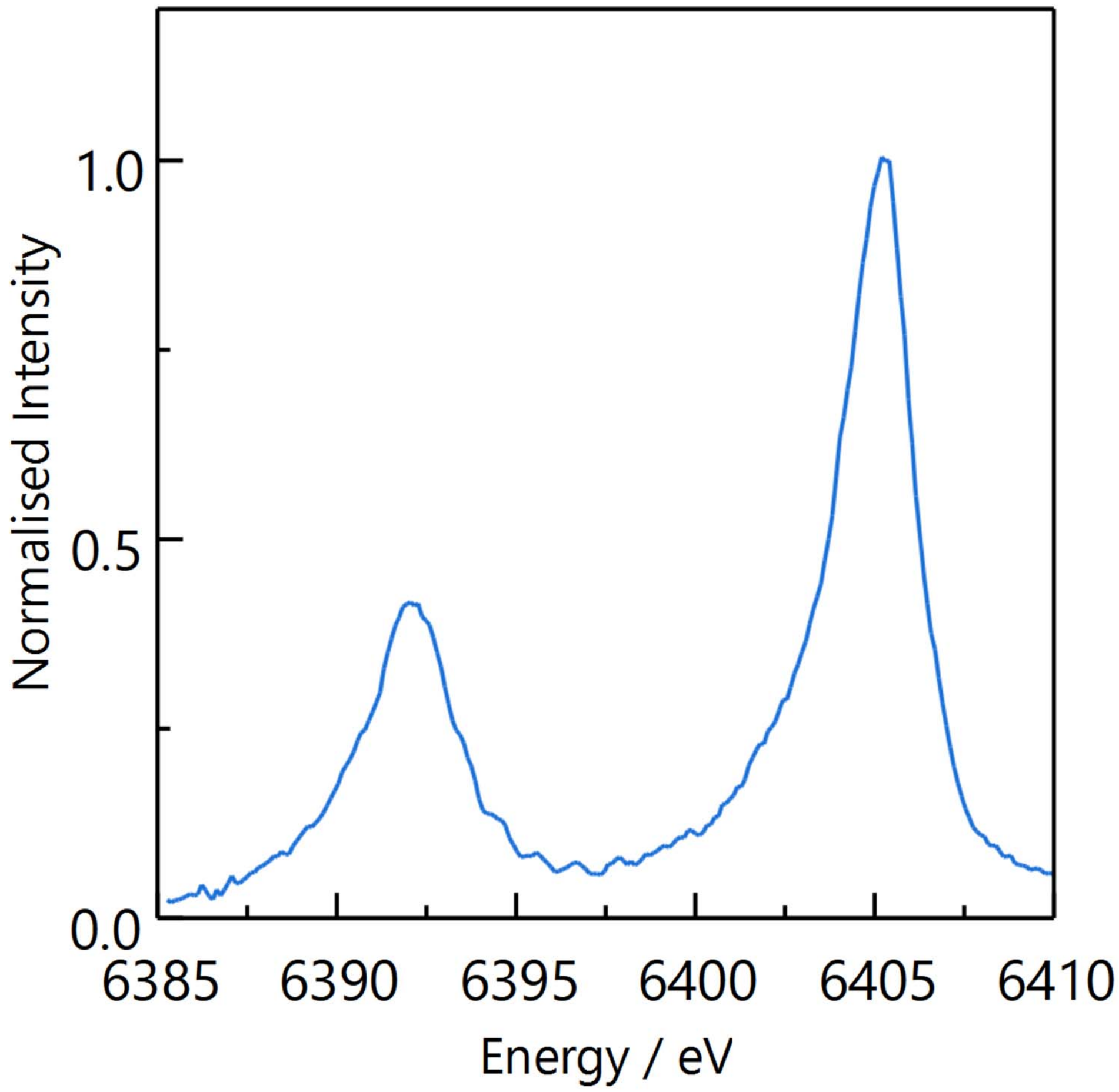

Figure S4 K$\alpha_{1,2}$ XES of an iron foil measured using the von Hamos prototype set up on VMXi. A three-point smoothing filter has been applied.

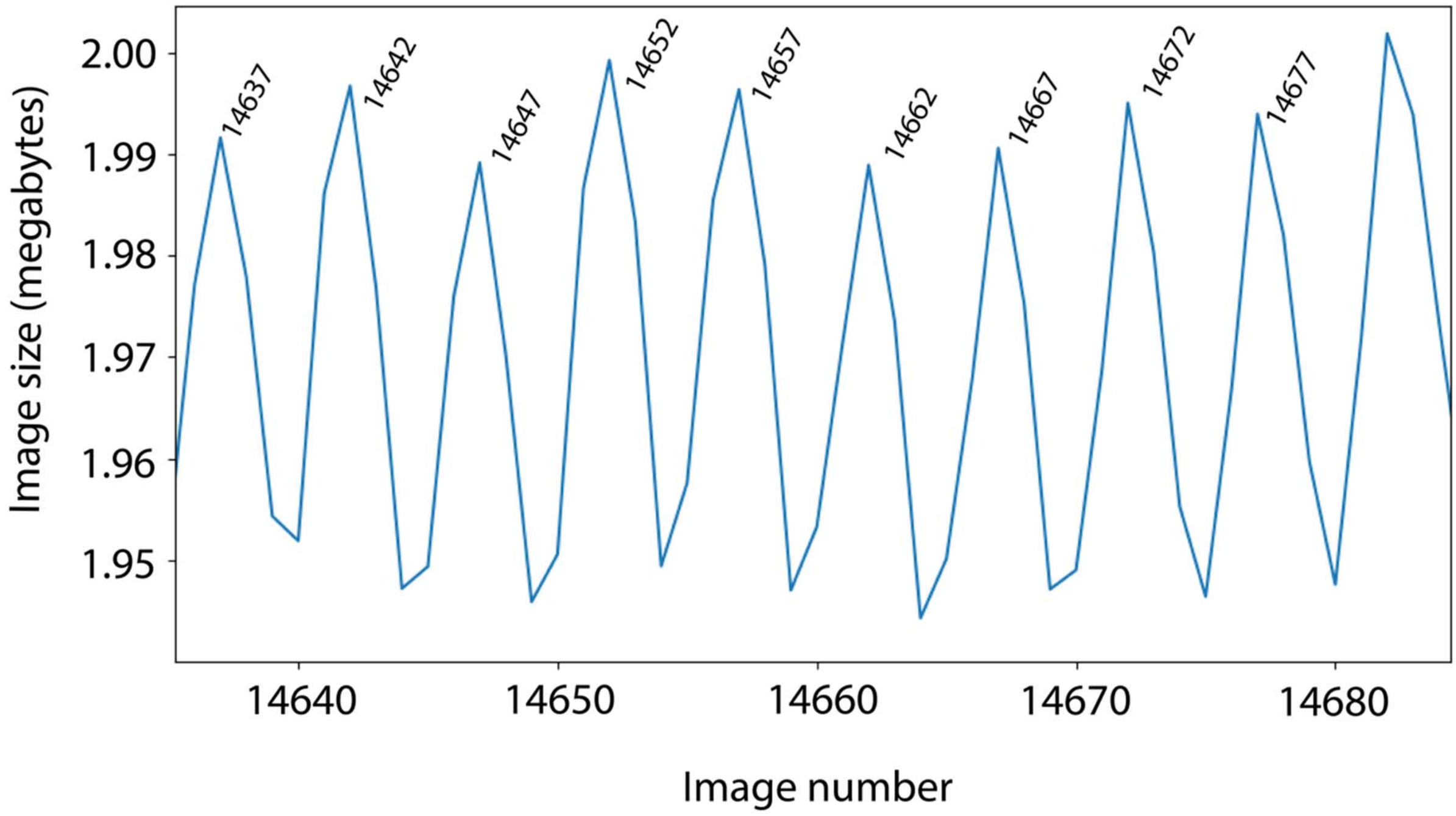

Figure S5 : Live feedback to monitor diffraction detector image size vs image number as a way to identify when a droplet is in the X-ray beam. Detector images were collected at 500 Hz but droplets were deposited onto the tape at 100 Hz. The image size is larger if/when a droplet is in the beam due to water scattering at low *q* regardless of whether or not the image also contains diffraction. A file size peak is observed every 5 images, which correlates with when a droplet is in the beam and diffraction if a crystal also intersects the beam. This serves as a useful proxy to aid synchronisation of droplets and diffraction data collection.

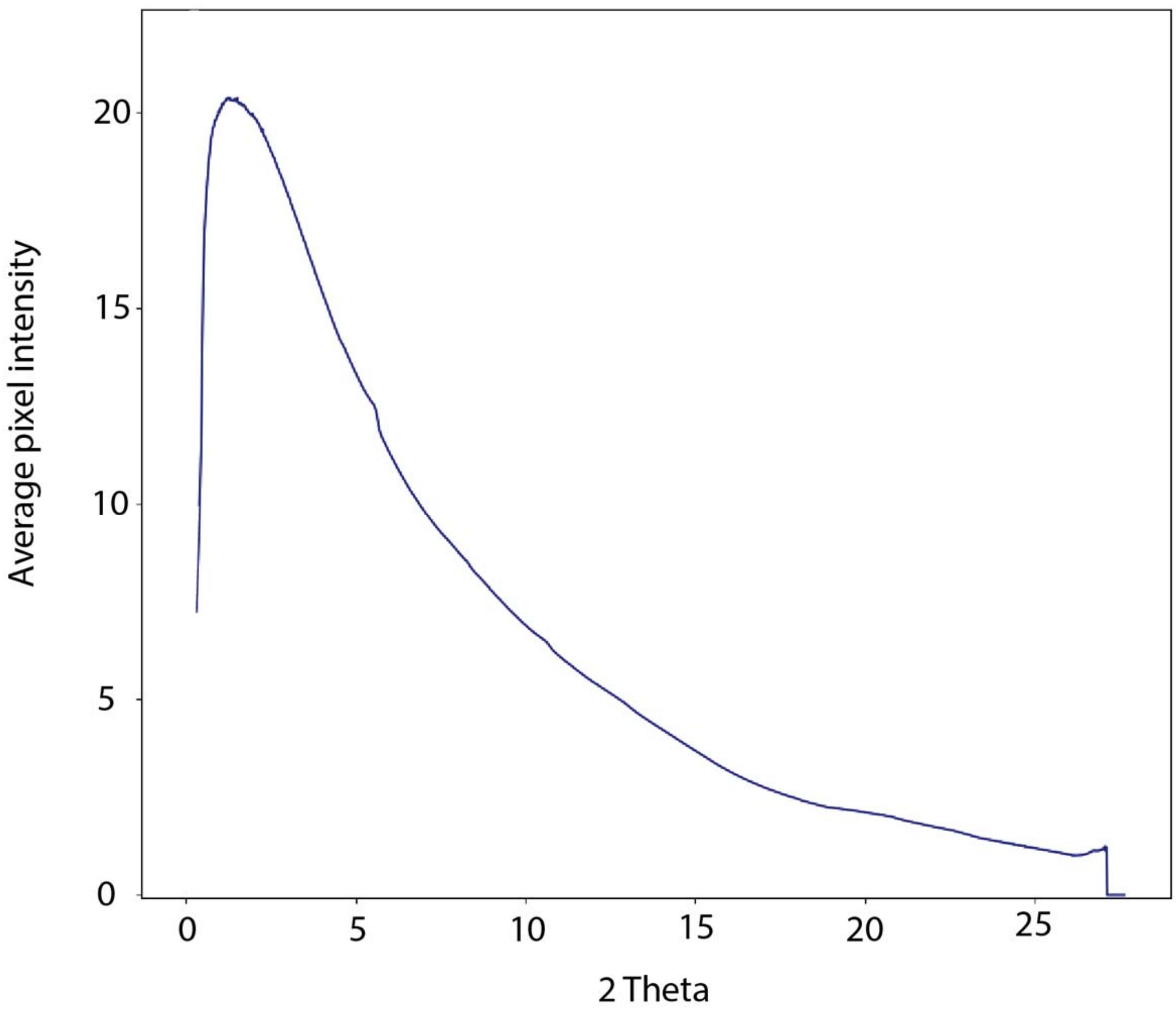

Figure S6 : Radial average from the calculated average image from the CTX-M-15 data set (~192,000 images).

Table S1 Comparison of sample efficiency for CTX-M-15 between SACLA and VMXi (Diamond).

	DoT (average over 3 data sets)*	Prototype tape drive VMXi
Volume of slurry per data set (µl) at ~ 10^8 crystals/ml	332 (300+420+275)	100
mg protein per data set	2.7 (2.4+3.4+2.2)	1.2
Total number of images collected	64,318 (59,137+63,778+70,040)	192,000
Number of lattices	11,864 (4,502+15,151+16,028)	20,556
Lattices/ µl slurry	35.7	214
Lattices/ mg protein	4,394	17,130

*From SACLA (Butryn et al., 2021).

The CTX-M-15 microcrystal slurry used at SACLA and Diamond has the same density ~ 10^8 crystals/ml.

Table S2 Multiple lattices among CTX-M-15 data set.

Total number of diffraction patterns / lattices	20556
Total number of images indexed	19980
Images containing 1 lattice	19406
Images containing 2 lattices	572
Images containing 3 lattices	2

Table S3 Signal to noise ratio (SNR) for the XES spectrum of each of the presented samples calculated using the Kα1and Kα2 signals.

	SNR	
Sample	$K\alpha_1$	$K\alpha_2$
$K_4Fe(CN)_6$	620	242
$K_3Fe(CN)_6$	1179	479
AcNiR	34	15
Cu(I)Cl	1953	845
$Cu(II)Cl_2$	2043	899
